# Multi-actor collaborations in primary health care implementation: a Social Network Analysis of the primary health care strategy in Ghana

**DOI:** 10.1093/heapol/czag027

**Published:** 2026-02-25

**Authors:** Dominic Dormenyo Gadeka, Patricia Akweongo, Genevieve Cecilia Aryeetey, Eleanor Beth Whyle, Justice Moses K Aheto, Lucy Gilson

**Affiliations:** Department of Health Policy, Planning and Management, University of Ghana School of Public Health, P.O. Box LG13, Legon, Accra, Ghana; Department of Health Policy, Planning and Management, University of Ghana School of Public Health, P.O. Box LG13, Legon, Accra, Ghana; Department of Health Policy, Planning and Management, University of Ghana School of Public Health, P.O. Box LG13, Legon, Accra, Ghana; Division of Health Policy and Systems, School of Public Health and Family Medicine, University of Cape Town, Anzio Road, Observatory 7925, South Africa; Department of Biostatistics, University of Ghana School of Public Health, P.O. Box LG13, Legon, Accra, Ghana; Division of Health Policy and Systems, School of Public Health and Family Medicine, University of Cape Town, Anzio Road, Observatory 7925, South Africa; Department of Global Health and Development, London School of Hygiene and Tropical Medicine, Keppel Street, London WC1E 7HT, United Kingdom

**Keywords:** Social Network Analysis, actor networks, primary health care, policy implementation, Ghana, low- and middle-income countries

## Abstract

Bottom-up theory demonstrates that networks of actors play important roles in policy implementation, yet limited attention has so far been paid to the influence that actor networks might have on the implementation of primary health care (PHC) strategies and outcomes. This study examined the roles actor networks play in the implementation of Community-based Health Planning and Services (CHPS) in Ghana, focusing on the nature and patterns of relations and structure and strength of prevailing collaborations. This was a cross-sectional study using a social network analysis methodology in eight districts across two regions in Ghana. The study population was implementers of CHPS from the community, district, regional, national, and development partners. Data were obtained using a modified pretested closed-ended social network questionnaire. To establish collaborative relationships, knowledge of other actors and the degree of communication on issues related to CHPS implementation were surveyed. Data were analysed using Gephi software version 0.9.2. The analysis demonstrated existing actor networks of Community Health Committees (CHCs), Community Health Officers (CHOs), Community Health Volunteers (CHVs), Sub-district, and district-level networks, including local government actors and political leaders, as well as regional, national, and development partner actors in CHPS implementation. The nature of relations showed isolated networks of CHCs, CHVs, and sub-districts across both regions. Patterns of interactions revealed that CHO networks collaborate with each other, while CHCs primarily collaborate with CHOs. Overall, weak collaborative relationships were noted among the actor networks (network density <10%). The results suggest segmented, decentralized networks with limited involvement of critical actors, including community-level, local government, political leaders, national-level, and development partners in CHPS implementation. The network analysis highlights weak collaborative relationships among actor networks in CHPS implementation, a practice which negatively impacts its implementation experience. The study highlights pathway to strengthen cohesion and improve collaborative relationships in addressing CHPS as a PHC strategy.

Key messages

- This study contributes to empirical literature from low- and middle-income countries that explains how actor networks (set of individuals who interact on a given policy issue) influence primary health care (PHC) implementation
- Weak collaborative relationships among actor networks negatively impact Community-based Health Planning and Services implementation as a PHC strategy in Ghana
- Strengthening cohesion and improving collaborative relationships among actor networks involved in PHC implementation may strengthen implementation in addressing PHC goals.
- Social Network Analysis demonstrates itself as a useful tool as it offers value in generating policy-relevant theoretical insights about networks and their influences on policy implementation

## Introduction

The goal of primary health care (PHC) is to bridge the health equity gap by ensuring that all people, everywhere, have access to the right care in their community (World Health Organization 2018). Evidence shows PHC as a cost-effective strategy to sustain health and health systems gains and the foundation on which Universal Health Coverage (UHC) and the health-related Sustainable Development Goals (SDGs) can be achieved (World Health Organization 2018). However, the implementation of PHC strategies in most low- and middle-income countries (LMICs) remains weak (Bitton et al. 2019).

In 2000, Ghana operationalized its ‘bottom-up’ PHC strategy, the Community-based Health Planning and Services (CHPS) strategy, to improve equity in access, efficiency, and responsiveness to clients’ needs and to develop effective intersectoral collaboration (Ghana Health Service 2005, Nyonator et al. 2005). CHPS is the mobilization of community leadership, decision-making systems, and resources in a defined catchment area (zone) and involves the placement of reoriented frontline health staff (Community Health Officers—CHOs) with logistics support and community volunteer systems to provide services according to the principles of PHC (Ghana Health Service 2005). CHPS demands systematic planning and execution by critical actors from the community to the national level, including development partners (Ghana Health Service 2004, World Health Organization 2017). However, more than two decades after its implementation, the CHPS strategy continues to face challenges despite adapting service delivery and extending coverage as efforts in addressing the bottlenecks experienced (Awoonor Williams et al. 2019, Appiah-Agyekum 2020, Ministry of Health 2020). For instance, poor health infrastructure and referral systems, weak intersectoral actions, shortage and uneven distribution of skilled health professionals, negative attitudes of health professionals, inadequate logistics, and health commodities, as well as inefficient allocation of available resources, remain key bottlenecks (Ministry of Health 2016, 2020, Kweku et al. 2020a, 2020b). In addition, the governance systems, particularly the linkages between the interfaces of CHPS (community, sub-district, district, regional, and national levels, including development partners), are also deemed weak (Atinga et al. 2018, Kweku et al. 2020a).

While bottom-up theory demonstrates that networks of actors play important roles in policy implementation, this has not been explored in relation to the CHPS strategy. Bottom-up theorists argue that policy implementation involves complex interactions among networks of interdependent actors and that implementation success or failure depends on the extent of cooperation achieved among the involved actors (Hill and Hupe 2009). In addition, to strengthen implementation, bottom-up theory argues that there is a need to understand the goals, strategies, activities, and contacts of actors involved in the implementation process (Hanf et al. 1978, Hjern and Porter 1981). Furthermore, earlier work that mapped the existing body of actor network-related research in low- and middle-income settings shows that the existence and use of networks in PHC can improve performance (Gadeka et al. 2023). Understanding the roles that the networks involved in the CHPS implementation play may help generate ideas about how to strengthen CHPS as a PHC strategy. To this end, Social Network Analysis (SNA) has been applied in health policy analysis work in recent years to identify the nature and patterns of relations and the structure and strength of collaborative relationships among actors and/or their networks. SNA remains one of the ways to study collaborative interactions between health system actors and to provide an understanding of the behaviour of actors involved in a network and point to gaps in relationships that are required to strengthen the health system for collective action (Blanchet and James 2012, De Brún and McAuliffe 2018).

Networks can be categorized along multiple axes based on the level of social interactions: control (centralized vs. decentralized), connectivity pattern (integrated vs. segmented), and specialized forms (‘wheel/star,’ ‘polycephalous,’ ‘clique’, and segmented, decentralized networks) (Carrington et al. 2005, Scott and Carrington 2011). For centralized networks, one or a few central actors control communication, decision-making, and resource distribution. In contrast, decentralized networks distribute control and decision-making among multiple actors, with no single actor dominating (Carrington et al. 2005, Scott and Carrington 2011). In terms of integrated networks, there is high connectivity, where most actors within the networks are reachable from one another. This network type fosters rapid information flow and reduces fragmentation (Carrington et al. 2005, Scott and Carrington 2011). For segmented networks, the actors exist in semi-independent clusters with limited inter-cluster connectivity. The ‘wheel/star’ networks involve the connection between one central actor and multiple peripheral actors, with whom all communications pass, where peripheral actors do not communicate directly with each other (Carrington et al. 2005, Scott and Carrington 2011). Polycephalous networks are semi-decentralized with multiple central actors controlling distinct clusters, allowing parallel problem-solving, while ‘clique’ networks involve fully connected groups, mostly integrated and cohesive, where each actor has a direct connection to all others. Segmented, decentralized networks consist of multiple semi-autonomous clusters with decentralized control, encouraging local decision-making (Carrington et al. 2005, Scott and Carrington 2011).

This study, using SNA, examined the roles actor networks play in implementing CHPS, particularly the nature and patterns of relations and the structure and strength of prevailing collaborations to inform further policy design of CHPS.

## Materials and methods

### Study design

We conducted a cross-sectional study to identify the role of actor networks in CHPS implementation. Table 1 presents operational definitions of key network concepts and metrics used in the study. We applied Granovetter's (1973, 1983) ‘the strength of weak ties’ theory to explain the dynamics of collaboration.

**Table 1:** Operational definition of network concepts and metrics.

Network concepts/metrics	Definition
Node	An actor
Tie/edge	A knowledge of other actors or a communication link between actors
Actor	Individuals or organizations who influence CHPS implementation, either positively or negatively
Actor network	A set of individuals or organizations that interact on a given policy (CHPS implementation)
Isolates	A network or an actor that has no connection
Density	The number of ties a set of actors has in relation to the number of possible ties they can have
Broker/boundary spanner	An actor who connects (otherwise) disconnected networks
Degree centrality	The number of ties of an actor
Betweenness centrality	The number of times an actor lies on the shortest path between other actors
Centralization	The extent to which a set of actors are organized around a central actor
Bridges	Actors who facilitate information sharing to reach isolated networks or actors
Network structure	The cohesion or connectedness of the network, depicting density or fragmentation of the network
Network shape	The distribution of ties between actors

### Study setting

**Figure 1:**
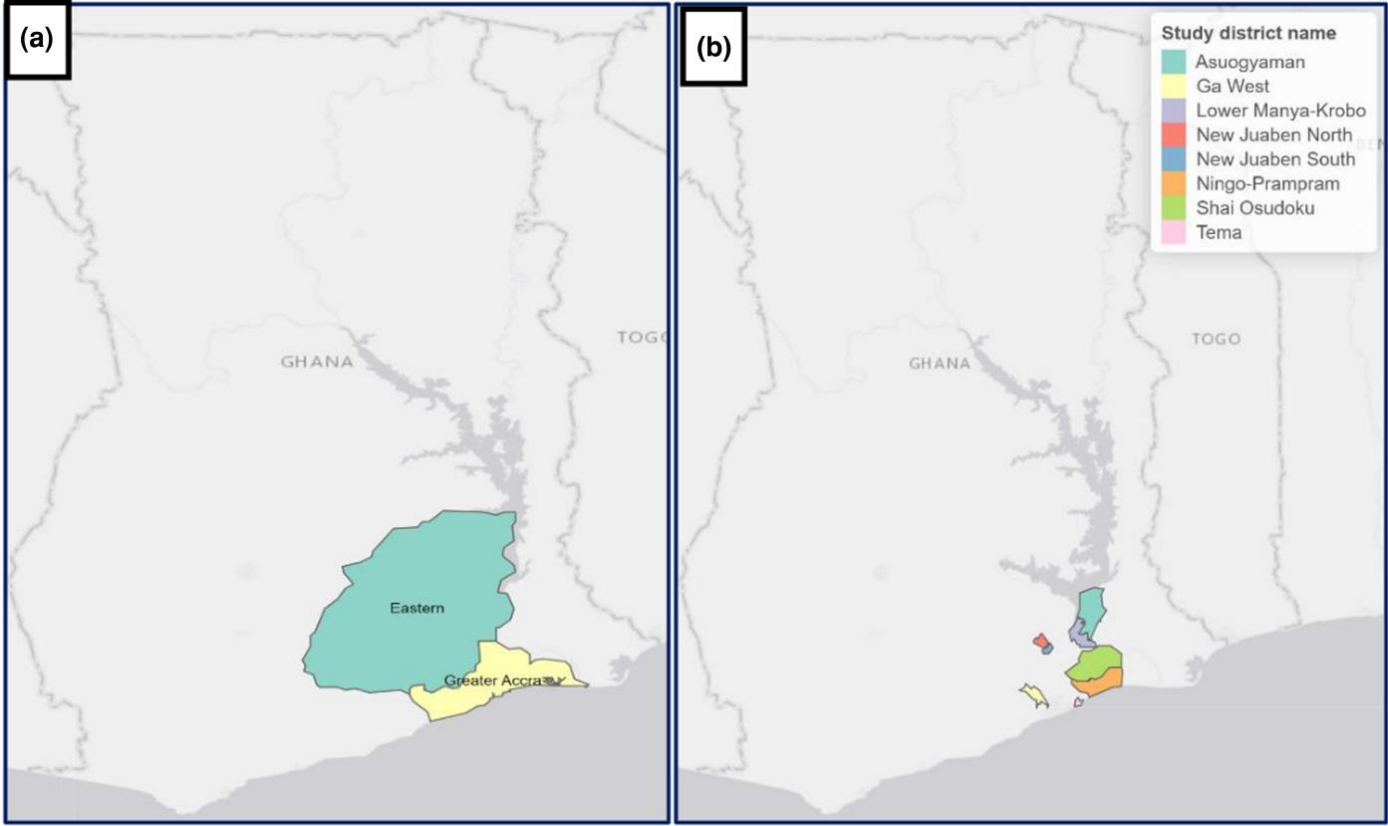
Map of Ghana showing the study regions (a) and districts (b) (source: produced by authors in R).

The study was carried out in the Greater Accra and Eastern regions of Ghana (Fig. 1). The Greater Accra region shares a boundary with the Eastern region to the north, and it is the most urbanized region in Ghana. The two regions were purposively selected to represent the coastal/southern belts of Ghana and to provide a better context to understand the challenges confronting CHPS to enable policy design and reform that would help in its implementation. The coastal regions are noted for the multiplicity of healthcare facilities and services which could be contributing factors to the constraints and bottlenecks that currently characterize the CHPS implementation. Four districts were purposively selected from each of the two regions. The districts selected included only those with CHPS compounds. The Ga West municipality, Ningo-Prampram municipality, Shai-Osudoku municipality, and Tema Metropolitan area were selected in the Greater Accra region while the Asuogyaman municipality, New Juaben South municipality, New Juaben North municipality, and Lower Manya Krobo municipality represented the selected districts in the Eastern region. Selecting eight districts sought to provide adequate network sizes and structures to enable sound analyses of the study.

### Study population

The study population was actors spanning the community to the national level. These include community health committees (CHCs), community health volunteers (CHVs), CHOs, Sub-District Health Management Teams (SDHMT), District Health Management Teams (DHMTs), Regional Health Management Teams (RHMTs), national-level actors and development partners who were involved in the CHPS implementation. To enable the collection of rich data to answer the study objective, only actors who were involved in the CHPS implementation over the last 6 months or more were included in the study. The study was conducted between December 2021 and April 2022.

### Data collection

Data were obtained using a modified pretested closed-ended social network questionnaire (Supplementary material) (Mukinda et al. 2022). We sought to include all actors in each district. The questionnaire was pretested in a similar but nonstudy district to ascertain the validity and reliability within the Ghanaian context. Data collection followed the sequence of steps suggested by Blanchet and James (2012). These are detailed below.

#### Identifying and describing relevant actors for the network

First, the actors were identified following a ‘roster’/name generator approach (recall list-to identifying respondents) (Blanchet and James 2012). To do this, initial lists of actors involved in the CHPS implementation were obtained from CHPS compound in-charges, sub-district leaders, district CHPS coordinators (DCHPSs), and assemblymen of the various districts, sub-districts, and communities. The names were collated for each district and region before the data collection. The community-level actors included CHCs, CHVs, CHOs (nurses/midwives/officers), sub-district heads, and field technicians/disease control officers. The district and regional-level actors included the DHMT members, RHMT members, National Health Insurance Authority (NHIA) officers at the districts, Members of Parliament (MPs), and staff of Metropolitan, Municipal and District Assemblies (MMDAs). The national-level actors were from the Ministry of Health (MOH), Ghana Health Service (GHS), Expanded Programme on Immunization (EPI), and development partners/Civil Society Organizations.

To establish the networks among the actors, the respondents (egos) were presented with the cumulated list of the names (alters) from which they were asked to select those they knew. Respondents were allowed to add any missing names to the list during the survey.

#### Defining meaningful relationships between actors

The second step involved the definition of meaningful relationships between the actors. Meaningful network relationships were defined as those relationships that facilitated action or decision-making among the actors (Cross and Parker 2004, Mukinda et al. 2022). A relationship was reported to exist when the respondent (ego) stated it. The reporting of the relationship, therefore, did not depend on both the respondent and alter (other actors) indicating its existence. To establish the collaboration networks, knowledge of other actors involved in the CHPS implementation was considered as a prerequisite, while the degree of communication on issues related to the CHPS implementation was considered a type of collaborative relation (Cross and Parker 2004). Meaningful collaborative interactions were therefore represented by the knowledge of other actors and the degree of communication.

The degree of communication was measured using a five-point Likert scale. The respondents were asked to rate their level of interactions with other actors by stating their frequency of communication with these actors on issues regarding CHPS implementation by choosing the corresponding number as follows (0 = never, 1 = once a quarter or beyond, 2 = monthly, 3 =weekly, and 4 = daily).

The second part of the questionnaire explored the background characteristics of the respondents, which included the sex of the respondent, the age, their current position, and duration in that position as a CHPS implementor.

### Data analysis

#### Visualization of the network structure and position of actors

Data were entered into Microsoft Excel^®^ 2019, managed, and then imported to Gephi software version 0.9.2 for network visualization and analysis (GEPHI 2015). To ensure compatibility with Gephi software version 0.9.2, the Excel matrices of the data were saved as comma-delimited values (.csv). Graphs (sociograms) were then generated for each actor network and region in Gephi. The network graphs mapped the different categories of actors from the community to national, including the development partners/civil society organizations who were involved in the CHPS implementation. The networks structure and shape were examined. Additionally, the roles and positions of actors in the network, which were characterized as central highly connected actors and peripheral actors with loose ties (Mukinda et al. 2022), were explored.

#### Description of the network structure

In the sociogram, actors were represented by nodes (coded circles) and relations between actors were denoted with lines (edges). The size of the node represents the number of connections (degree centrality) or the frequency of communication between the actor and others on issues regarding CHPS implementation. Different colours were used for the different actor network categories and their positions in the network. The lines move from the egos (the responding actors) to alters, with the ego node matching the colour of the line. Arrowheads were used to indicate the direction of the flow of the link/relation (representing directed ties). An arrow with two heads indicates actors with a mutual exchange of links. Where two actors exchange more than one link, arrowheads with different colours were used to represent the links. Links without arrows represent undirected ties between the actors. For instance, links established through the frequency of communication between actors in this study were undirected ties and therefore were represented without arrowheads.

## Results

### Characteristics of study respondents

Of 130 respondents (actors) who participated in the survey (Table 2), more than two-thirds (102, 78.5%) were aged 25–49 years and over 50% were females. More than half of the actors (70, 53.9%) were CHOs, while 37 (28.5%) were CHC members. Most of the actors (84, 64.6%) had been involved in CHPS implementation for 5 years, while more than two-thirds had been in their current position over the past 5 years.

**Table 2:** Characteristics of study respondents.

Characteristics	Greater Accra	Eastern region	Total
	*N* (%)	*N* (%)	*N* (%)
Age			
25–49 years	53 (85.25)	49 (72.73)	102 (78.46)
50+ years	10 (14.75)	18 (27.27)	28 (21.54)
Sex			
Male	26 (42.62)	31 (46.97)	57 (43.85)
Female	37 (57.38)	36 (53.03)	73 (56.15)
Level of work			
Community level	17 (27.87)	20 (30.30)	37 (28.46)
CHPS compound (CHOs)	34 (55.74)	36 (54.55)	70 (53.85)
Sub-district	5 (6.56)	4 (4.55)	9 (5.38)
District	7 (9.84)	7 (10.61)	14 (10.00)
Years involved in CHPS implementation			
1–5	41 (67.21)	43 (65.15)	84 (64.62)
Above 5	22 (32.79)	24 (34.85)	46 (35.38)
Number of years in current position			
1–5	48 (75.41)	53 (78.79)	101 (77.69)
Above 5	15 (24.59)	14 (21.21)	29 (22.31)
Districts			
Asuogyaman	0 (0.00)	18 (26.87)	18 (13.85)
Ga West	17 (26.23)	0 (0.00)	17 (13.08)
Lower Manya Krobo	0 (0.00)	15 (22.73)	15 (11.54)
New Juaben North	0 (0.00)	15 (21.21)	15 (11.54)
New Juaben South	0 (0.00)	19 (28.79)	19 (14.62)
Ningo-Prampram	13 (19.67)	0 (0.00)	13 (10.00)
Shai-Osudoku	16 (26.23)	0 (0.00)	16 (12.31)
Tema Metro	17 (27.87)	0 (0.00)	17 (13.08)

In the eight districts, the actors were fairly spread across districts, with each district accounting for 10%–15% of actors. Similarly, the actors were fairly spread across the two regions. In addition, ∼37 (57.4%) and 36 (53%) of the actors from the Greater Accra and the Eastern regions, respectively, were females. In both regions, the majority of health staff, 34 (55.7%) in Greater Accra and 36 (54.6%) in Eastern, were from the CHPS compound level (CHOs). Also, more than two-thirds of health staff, 41 (67.2%) and 43 (65.2%) of the actors from the Greater Accra and Eastern regions, were involved in CHPS implementation over the last 5 years (Table 2).

### Actor networks identified

**Figure 2:**
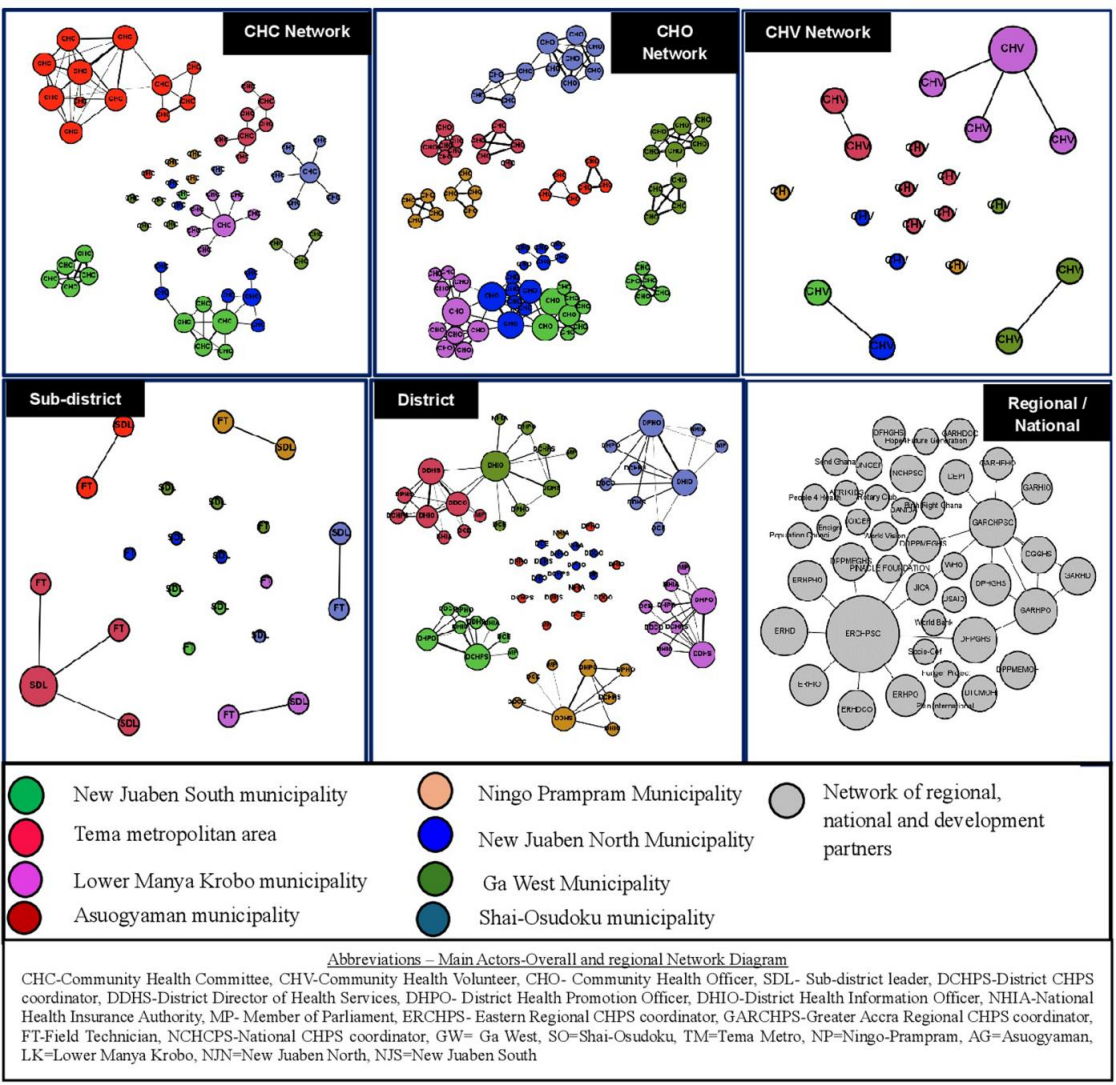
Existing actor networks—‘I know this person’ (knowledge network) across districts and regions.

The analysis demonstrated existing actor networks in CHPS implementation. Figure 2 shows the actor networks identified in the study. The study highlights actor networks of CHCs, CHOs, CHVs, SDHMTs, district-level networks including DHMTs and MMDAs, and network of regional, national, and development partners as existing networks.

### Nature of relations among actor networks

Across the existing actor networks, observable relationships were identified. The study shows that out of the 130 actors who participated, a total network comprising 303 actors (nodes) and 2503 connections across all levels (community, sub-district, district, regional, national, and development partners) was identified in the two study regions. We present the findings by region, district, sub-district, and community levels in the sections below.

#### Region

The results showed that the overall networks of actors involved in CHPS implementation in the two regions were loosely connected, with an average density of <0.1 (Table 3), suggesting weak relationships among the actors. The Eastern region recorded a relatively higher network density (0.08), suggesting a better relationship among actor networks compared with the Greater Accra region density of 0.07.

**Table 3:** Network metrics across eight CHPS implementation districts.

Regions	Network level	Network density	Average degree	Network diameter
Eastern region	New Juaben South	0.11	15.03	3
	Asuogyaman	0.10	7.90	4
	New Juaben North	0.08	6.18	5
	Lower Manya Krobo	0.14	10.21	4
	Overall network	0.08	13.94	4
Greater Accra region	Shai-Osudoku	0.11	7.87	4
	Ga West	0.09	6.89	5
	Tema Metro	0.08	5.73	5
	Ningo-Prampram	0.09	5.80	5
	Eastern region	0.08	13.94	4
	Overall network	0.07	11.20	7

#### District

The district networks consisted of the district health information officer (DHIO), district director of health services (DDHS), DCHPS, district public health officer (DPHO), district health promotion officer (DHPO), district disease control officer (DDCO), MP, NHIA at the district level, and district/municipal/metropolitan chief executive (DCEs) or the district assemblies. Apart from Asuogyaman and New Juaben South municipalities, no relationships were observed among district networks across the other districts (Fig. 2). The Lower Manya Krobo municipality had the highest network density, 0.14, while the Tema Metro recorded the least, 0.08 (Table 3), demonstrating a more closely connected (more integrated) network compared with all other districts.

#### Sub-district

The sub-district networks consisted of the sub-district leader and the sub-district field technician/disease control officer. There were no relationships between sub-district networks across districts (Fig. 2).

#### Community level

The community-level networks included CHCs, CHVs, and CHOs. In each region, the CHC networks showed segmented networks in the districts (Fig. 2). That is, there was no relationship between CHCs across districts. The CHC networks in the Eastern region showed a better relationship among themselves compared with the Greater Accra region, except in the Tema metropolitan area. Existing relationships of CHO networks were observed across districts in the Eastern region: Lower Manya Krobo, New Juaben South and North municipalities, while no relationships were observed in the Greater Accra region (Fig. 2). The networks of CHV were only found in the Eastern region, specifically, New Juaben North and South municipalities and with no relationships among themselves (segmented networks).

### Patterns of relationships of actor networks across regions and districts

**Figure 3:**
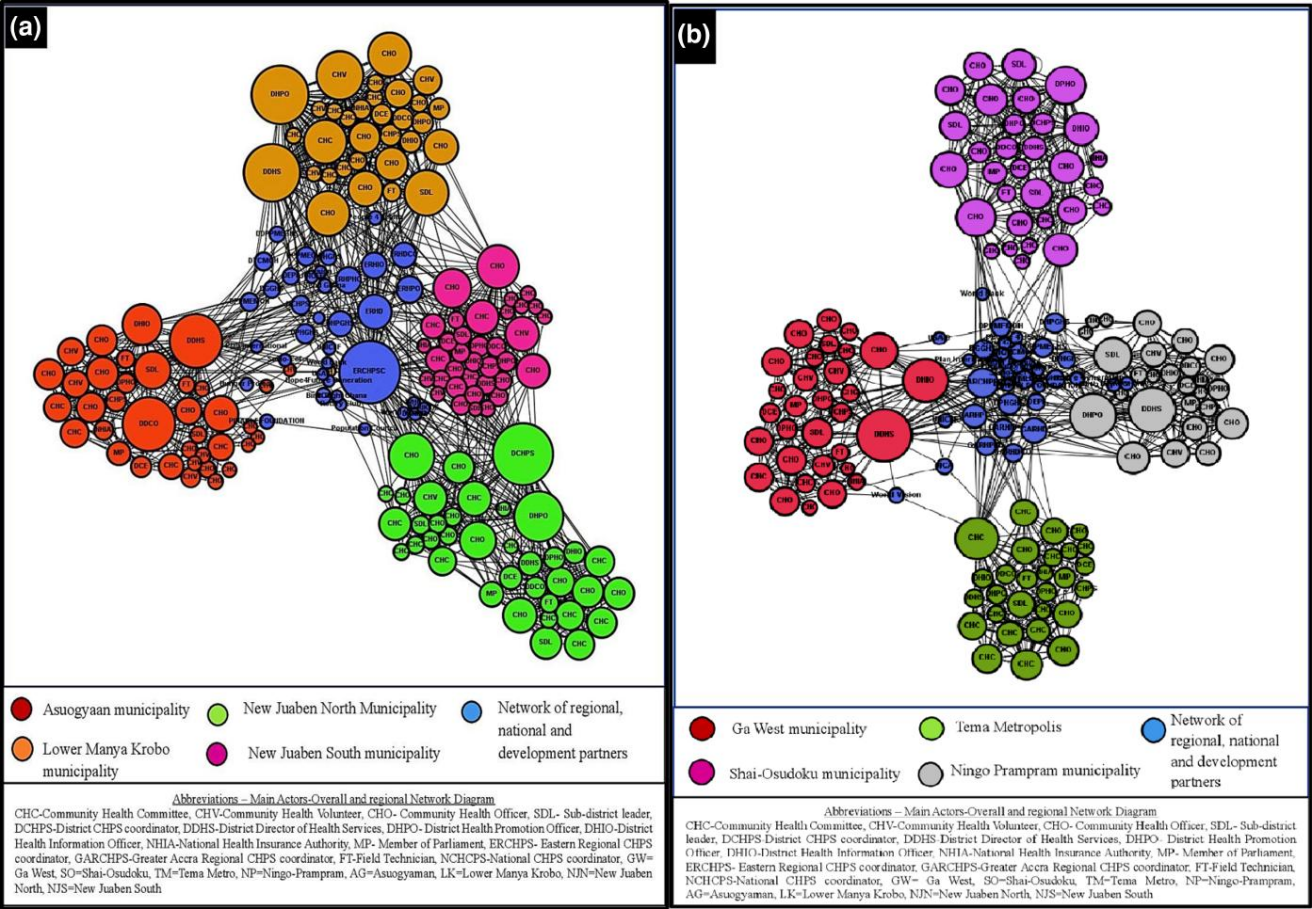
‘I know this person’ network in the Eastern region (a) and the Greater Accra region (b).

The analysis also revealed patterns of the relationships across the identified actor networks. The results showed that the most central actor (prominent/dominant actor) above the district interface was the Eastern regional CHPS coordinator, while the National CHPS coordinator represented the least central actor in the overall network. Additionally, the NHIA, MPs, District Assemblies (MMDAs), and development partners had a weaker relationship with the other CHPS implementors across the eight districts in the two regions (Fig. 3a and b). We present details of these patterns by region and district.

#### Region

The network structure (Fig. 3a) showed that the network for the Eastern region revolved around the different interfaces (community, CHPS compound, sub-district, and district): DDHS, DHPO, Sub-district leader (SDL), CHOs, CHVs, and CHCs compared with the Greater Accra region, which had less involvement of most of these actors (Fig. 3b).

#### District

For each district, the network structure showed that the dominant actors in the network with respect to central connectors and boundary spanners varied across the districts. In the Eastern region (Fig. 3a), the network in the Lower Manya Krobo Municipality revolved around across the different interfaces (community, CHPS compound, sub-district, and district): DDHS, DHPO, Sub-district leader (SDL), CHOs, CHVs, and CHCs while in the Tema Metropolis in the Greater Accra region (Fig. 3b), the most important actors in the network were the CHOs and CHCs.

### Structure and strength of collaborations of actor networks in the CHPS implementation

In terms of the structure and strength of collaborations of the actor networks in the CHPS implementation, the overall network showed segmented, decentralized networks with weak collaborative relationships (network density <10%) (Table 4). Details of the findings are presented in the following sections, organized by region, district, sub-district, and community levels.

**Table 4:** Network metrics of collaboration.

Region	Network level	Network density	Average degree	Average weighted degree	Network diameter
Eastern region	New Juaben South	0.07	6.00	20.39	4
	Asuogyaman	0.06	5.40	17.95	5
	New Juaben North	0.05	4.37	11.81	6
	Lower Manya Krobo	0.09	7.25	22.10	4
	Overall network	0.06	10.43	33.81	5
Greater Accra region	Shai-Osudoku	0.08	6.15	19.09	4
	Ga West	0.06	4.45	14.63	4
	Tema Metro	0.06	4.86	12.76	6
	Ningo-Prampram	0.05	3.55	12.08	5
	Overall network	0.05	8.57	27.16	6

#### Region

**Figure 4:**
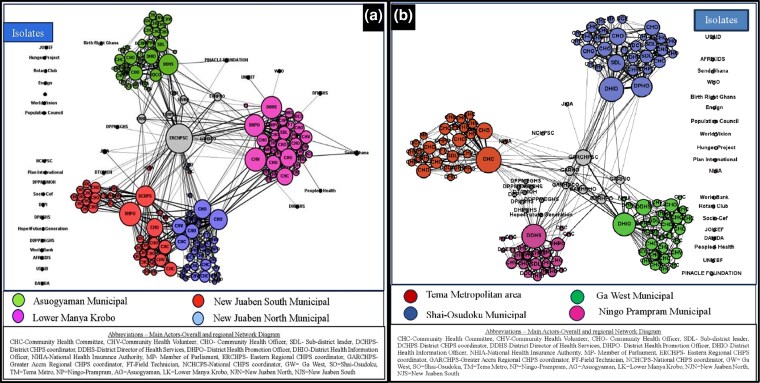
‘Degree of communication’ network (how often do you communicate) in the Eastern region (a) and the Greater Accra region (b).

The results showed a relatively better collaborative relationship among the different actor networks (CHCs, CHVs, CHOs, SDHMTs, district-level networks including DHMTs and MMDAs, and the combined network of regional, national, and development partners) in the Eastern (density = 0.06) (Fig. 4a) compared with the Greater Accra region (density = 0.05) (Fig. 4b), suggesting a stronger collaboration among CHPS implementors in the Eastern region compared with the Greater Accra region (Table 4). The results showed a stronger bridging or mediating role of the Eastern regional CHPS coordinator compared with the Greater Accra regional CHPS coordinator (Fig. 4a and b).

#### District

Across the districts, actor networks in the Lower Manya Krobo municipality (density = 0.09) of the Eastern region collaborated more often compared with any other district. The weakest collaboration was noted in the Ningo-Prampram municipality (density = 0.05) of the Greater Accra region (Table 4).

#### Sub-district level

The results showed no collaboration across sub-districts and districts (Fig. 4a and b). Sub-districts were linked through a combination of district-level actors, identified as central connectors or boundary spanners: DDHS, DHPO, DHIO, and DDCO.

#### Community level

Furthermore, the results showed that most of the collaborations occurred at the community level (segmented decentralized networks) in all districts (Fig. 4a and b). Patterns of collaboration across interfaces showed community health nurses tended to collaborate with each other, while CHCs members collaborated mostly with community health nurses.

### Key actors involved in the collaborative relationships across districts in the regions

The SNA also revealed key actors within the respective networks who are involved in collaborative relationships. The results showed that in the Lower Manya Krobo municipality of the Eastern region (Fig. 4a), the collaborative networks revolved around the DDHS, DHPO, CHO, SDL, CHV, and CHC members, while in the Tema metropolitan area of the Greater Accra region (Fig. 4b), the collaboration networks revolved around only the CHOs and the CHC members. For the remaining districts, there were variations in the position and role of the main actors around whom the network revolved, encompassing a mix of dominant actors. In the Ga West municipality, the main actors were the DDHS, DHIO, and the CHOs. In the Shai-Osudoku municipality, the main central actors were the CHO, DHIO, and DHPO. DDHS, DDCO, CHO, and SDL collaborated more to implement the CHPS in the Asuogyaman municipality, while DDHS, SDL, DHPO, and CHO were the main actors in the Ningo-Prampram municipality. In the New Juaben South, the collaboration network revolved around the DCHPS, CHO, and DHPO while the New Juaben North involves mainly CHOs and the CHC members.

The results suggest less involvement of critical actors, including district assemblies, political leaders, and volunteers in the CHPS implementation, indicating weak collaborations within the health sector and with key political and community actors. Additionally, the findings showed less collaboration between the health sector and the NHIA, the National CHPS coordinator, and development partners. These findings were more pronounced among districts in the Greater Accra region (network density = 0.05) compared with the Eastern region (network density = 0.06).

## Discussion

### Main findings of the study

The CHPS strategy in Ghana aims to enhance community involvement and ownership of PHC interventions and thus ensure equity in access to basic health services through effective intersectoral collaborations (Ghana Health Service 2005). This study examined the roles actor networks play in the implementation of CHPS, focusing on the nature and patterns of relations and the structure and strength of prevailing collaborations.

Our findings showed isolated networks of CHCs, CHVs, and sub-districts across the two clusters: Greater Accra and Eastern regions. The patterns of interaction revealed that CHO networks collaborate with one another, while CHCs primarily collaborate with CHOs. Overall, weak collaborative relationships (low density) were noted among the actor networks. The results suggest segmented, decentralized networks with limited involvement of critical actors, including CHVs, local government, political leaders, national-level, and development partners in CHPS implementation.

### Interpretation and implications of findings for CHPS implementation

These findings can explain why implementing the CHPS has been a challenge (Ministry of Health 2016, 2020, Kweku et al. 2020a, 2020b). The findings revealed that though the overall network was segmented, decentralized, only a few of the actors acted as central actors and had strong ties, who were embedded in a web of weak relations between them. The actors with the highest centrality were the district-level and community-level actors in the Eastern region. These central actors represented the brokers, sitting on the shortest path between other actors, facilitating connections and information flow between the different interfaces of CHPS, from the community to the national level, or translating and adapting the CHPS concept to local needs. There was thus a dependence on a few central actors, including the regional CHPS coordinator and a few of the district-level actors who play the role of connectors, bridges, or boundary spanners between actors. The low network density or connectedness of CHPS actors observed suggests a low level of cohesion, with fragmented and ineffective functionality (Haythornthwaite 1996). Increasing the density of ties among the disconnected actors and their networks will improve the efficiency of information diffusion between networks and strengthen implementation (Haythornthwaite 1996, Long et al. 2013).

The less-collaborative actors/networks, such as district assemblies, political leaders (MPs), and CHV networks in this study, represent weak ties in the overall network of CHPS implementors, yet they are expected to play critical roles in CHPS implementation. For instance, CHV networks, which were shown as characterized by weak ties in this study, were originally responsible for the mobilization and sensitization of communities to take action to manage health in the community, collaborate with the CHO, and support CHPS service delivery, assess and advise CHO networks on environmental factors in the home that can affect health, as well as assist the CHO networks in home visits (Ghana Health Service 2005). However, there is much evidence of low participation of CHVs in the CHPS implementation (Ministry of Health 2016, 2020, Kweku et al. 2020c). The failure of CHVs to perform their roles not only overburdens CHO and CHC networks, who try to augment these roles, but also negatively impacts the overall CHPS implementation (Ministry of Health 2016, 2020). Similarly, even though the district assemblies and MPs who represent the higher level of local government structure in the CHPS catchment areas are to support the construction and maintenance of CHPS compounds, provision of utility services such as safe water and electricity to the compound, assist in providing security to the CHPS compound as well as mobilize resources to support CHPS activities (Ghana Health Service 2005), they hardly do (Kweku et al. 2020b). The weaker involvement of these critical actors in the implementation of CHPS threatens not only the implementation of CHPS but also its sustainability.

### Implications of weak ties in CHPS implementation

The weak ties and isolated networks observed at all levels of the healthcare system, particularly at the sub-districts and by CHVs, can affect the overall CHPS implementation. This can explain the disconnect between the community and the healthcare system, in the form of low community participation and ownership of the CHPS reported in earlier studies (Kweku et al. 2020a, Ministry of Health 2020). In terms of sub-districts, the lack of collaboration could create undue pressure on the district-level actors who may find it difficult to respond timeously to the needs and demands of the sub-districts, CHPS compound, and communities. Moreover, PHC thrives on information, implying the need for stronger collaboration, particularly in the sub-districts. It is important that consideration is given to these weak ties. They represent an opportunity for innovation and for strengthening cohesion in a fragmented system (Mukinda et al. 2022). According to Granovetter (1973), ‘weak’ ties are acquaintances when compared with stronger ties of friendship or personal and professional support. Thus, when weak ties serve as bridges between different segments of a network, they become crucial in connecting structurally isolated clusters. They offer access to new information and resources. They shorten path distances and grant access to information unavailable within the immediate environment of the close-knit network (Klärner et al. 2022). By facilitating the dissemination of innovative ideas and promoting communication and collaboration between clusters, these ties enhance productivity and improve health outcomes. Additionally, Granovetter argues that weak ties offer the potential for ‘microintegration’, characterized by the regular exchange of information, or ‘macrointegration’, which facilitates episodic information sharing among fragmented networks, thereby strengthening the health system. Highlighting these weak ties, they could be strengthened and leveraged to serve as bridges connecting disparate actor networks in the CHPS implementation. This can take the form of re-engagement and education on CHPS objectives and their roles in the success of the implementation. Earlier studies on collaboration among actors in PHC implementation in LMICs found that effective collaboration facilitates information and resource sharing, enables high professional trust among actors, facilitates interactions among actors, and enables organizations to leverage for faster communication and resource flow (Kawonga et al. 2015, Ssengooba et al. 2017, Hall et al. 2019, Soi et al. 2020).

### Implications of the homogeneity of community-level collaborations

The findings also showed that most of the collaborations occurred at the community level among the CHO and CHC networks in all districts in each of the regions. Patterns of collaboration across CHPS interfaces show that CHOs tend to collaborate more among themselves. Though this could facilitate advice sharing and supportive supervision among the CHO networks (Assegaai and Schneider 2019, Karuga et al. 2019, Sabot et al. 2020), it has been suggested that networks are more likely to be successful in terms of their goals or policy intent when the network contains a diversity of actors from different professions, organizations, and sections of society (Sandström and Carlsson 2008). This is because the heterogeneity of actors is likely to bring more bridging opportunities and it is noted that a ‘high-performing actor network is a heterogeneous network with a high level of network density’ (Sandström and Carlsson 2008). Hence, a more integrated network of all relevant actors/networks in a collaborative decision-making process of CHPS is likely to enhance its implementation.

### A model multi-actor collaborative relationship to strengthen primary health care implementation

The study provides evidence that actor networks across the different health system levels play a role in CHPS implementation, offering empirical support for the networks highlighted in bottom-up policy implementation theory (Hanf et al. 1978, Hjern and Porter 1981). In this study, while the baseline demographic characteristics across the two clusters (Greater Accra and Eastern region) seem to be similar, our findings showed that the Eastern region appeared to provide a model of multi-actor collaborative relationship to strengthen PHC implementation. The model involves the following attributes:

- First, the regional-level leadership provided by the Eastern regional CHPS coordinator enabled stronger collaboration across districts by serving as a central actor, facilitating connections and information flow, and also serving as a bridge between the PHC system and the development partners and national-level actors.
- Second, the networks in the Eastern region revolved around the different interfaces: community (CHCs and CHVs), facility level (CHOs), sub-district, and district, demonstrating more integrated networks in the CHPS implementation. This enables stronger cohesion and facilitates the flow of information and other resources. It further prevents dependence on a few central actors who may become overburdened, consequently, negatively affecting the implementation.
- Third, the collaboration is driven by a multi-disciplinary team of actors: District Director of Health Service (DDHS), DHPO, Sub-district leader (SDL), CHOs, CHVs, and CHCs members, bringing together complementary skills and capabilities which are important for a functional PHC.
- Fourth, a better involvement of local government structure (district assemblies and MPs), demonstrating potential for stronger collaboration between the health and nonhealth sector actors.
- Lastly, there is a stronger level of involvement of community-level networks: CHOs, CHVs, and CHCs, indicating more decentralized networks which may contribute to community participation and ownership. This level of collaboration would avoid dependence on district networks for things that require local solutions.

The model demonstrates a more integrated collaborative relationship among actor networks. Overall, it speaks to the three interrelated and synergistic components of PHC: (i) individual, household/families and community empowerment for increased participation and enhanced self-care and self-reliance in health, demonstrated by the better involvement of community-level networks, (ii) comprehensive integrated health services that embrace primary care as public health goods, shown by the higher collaboration among the different networks, and (iii) multi-sectoral policies and actions to address wider determinants of health, demonstrated by the collaborative relationship between the local government structures and health sector actors (World Health Organization 2019).

## Study limitations

A limitation of this study is that it did not directly explore the linkage between the actor networks and the CHPS performance of the health system such as responsiveness. Additionally, the social network data may not have captured the dynamic nature of the relations/interactions between the actor networks involved in the CHPS service provision because it was cross-sectional. However, the use of the SNA accurately identified the actor networks, mapped, and measured the collaborative relationships between the networks and among the individual actors in providing an understanding of the roles of the networks involved in the CHPS implementation.

## Conclusions and recommendations

The network analysis revealed weak collaborative relationships among actor networks involved in the CHPS implementation, a practice which negatively impacts its implementation. Efforts at strengthening collaborative relationships, including re-engagement and education of CHPS implementors on its objectives and their roles in its successful implementation, will enhance cohesion, improve integrated actions, and strengthen its implementation. The use of SNA demonstrates itself as a useful tool as it offers value in generating policy-relevant theoretical insights about networks and their influences on policy implementation.

## Supplementary Material

czag027_Supplementary_Data

## Data Availability

The datasets used and/or analysed during the current study are available from the corresponding author on reasonable request.

## Reflexivity statement

The group of authors included four females and two males. The group comprised researchers based in Ghana and South Africa, with multiple levels of seniority from postdoctoral research fellow to associate and full professors. All authors have extensive experience in health systems and policy research. The authors’ areas of study varied and include health system strengthening, analysis of health policy and its implementation in LMIC health systems, health economics, health services, and qualitative and quantitative methods. These different experiences and perspectives were harnessed and brought to bear on the work reported in this paper.
