# Supplementary material for: Multi-actor collaborations in primary health care implementation: a Social Network Analysis of the primary health care strategy in Ghana
Source: Health Policy Plan. 2026 Feb 25;41(4):672–83. doi: 10.1093/heapol/czag027 (PMC13089405; doi:10.1093/heapol/czag027)
Supplement: czag027_Supplementary_Data [file czag027_supplementary_data.docx]

## **Supplementary file: Social Network Analysis Survey**

**Title:** Multi-Actor Collaborations in Primary Health Care (PHC) Implementation: A Social Network Analysis of the PHC Strategy in Ghana

DISTRICT CODE: _____________________________________

DISTRICT NAME: ____________________________________

REGION: ___________________________________________

Date of Interview: ___/____/______

**Introduction**

This study seeks to examine the role actor networks play in the implementation of CHPS as PHC strategy in Ghana. This section will map the networks for communication, information, problem solving, advice, and financial or other tangible resource supports among actors involved in the CHPS implementation in Ghana. The services provided through the CHPS strategy include vaccination/immunization, screening, prenatal/postnatal (antenatal), family planning, services for minor ailments (malaria, fever, diarrhea, acute respiratory infections and injuries etc), emergency deliveries, health education, counselling and nutrition services. The use of the social network will help to visually present the relationships between actors and the networks they form. Provision of answers to the questions in this section will take about 20 minutes of your valuable time to complete.

Your answers to the questions in this first part will allow us to map the network of communication in your District

| Q1. Name of Respondent |
| --- |
|  |

| Q2. At which level do you work in implementing the CHPS strategy in this district? | *[x]* |
| --- | --- |
| - National |  |
| - Regional |  |
| - District |  |
| - Sub-district |  |
| - CHPS/community level |  |
| - Development partner/International |  |

Q3. In this question, you are presented with a list of people involved in the Community-based health planning and services (CHPS) implementation in Ghana in this district from the national, regional, district, Sub-district and CHPS levels and you are requested to please mark with [X] the person you know (Q3)?

*[****Note:*** *Could you please add names of any person you know that is missing on the list. You can use the next page if you need more space]*

| **Q3: I know this person** *[Check all that apply]* | | | | | | | | | | | | | | |
| --- | --- | --- | --- | --- | --- | --- | --- | --- | --- | --- | --- | --- | --- | --- |
| **National** | |  | **Regional** | | **District** | | |  | **Sub-district** | | **CHPS/community level** | |  |  |
|  |  |  |  |  |  |  |  |  |  |  | |  |  |  |
|  |  |  |  |  |  |  |  |  |  |  | |  |  |  |
|  |  |  |  |  |  |  |  |  |  |  | |  |  |  |
|  |  |  |  |  |  |  |  |  |  |  | |  |  |  |
|  |  |  |  |  |  |  |  |  |  |  | |  |  |  |
|  |  |  |  |  |  |  |  |  |  |  | |  |  |  |
|  |  |  |  |  |  |  |  |  |  |  | |  |  |  |
|  |  |  |  |  |  |  |  |  |  |  | |  |  |  |
|  |  |  |  |  |  |  |  |  |  |  | |  |  |  |
|  |  |  |  |  |  |  |  |  |  |  | |  |  |  |
|  |  |  |  |  |  |  |  |  |  |  | |  |  |  |
|  |  |  |  |  |  |  |  |  |  |  | |  |  |  |
| Other (Specify) |  |  | Other (Specify) |  |  | Other (Specify) |  |  | Other (Specify) |  | | Other (Specify) |  |  |
| Other (Specify) |  |  | Other (Specify) |  |  | Other (Specify) |  |  | Other (Specify) |  | | Other (Specify) |  |  |
| Other (Specify) |  |  | Other (Specify) |  |  | Other (Specify) |  |  | Other (Specify) |  | | Other (Specify) |  |  |
| Other (Specify) |  |  | Other (Specify) |  |  | Other (Specify) |  |  | Other (Specify) |  | | Other (Specify) |  |  |
| Other (Specify) |  |  | Other (Specify) |  |  | Other (Specify) |  |  | Other (Specify) |  | | Other (Specify) |  |  |
| Other (Specify) |  |  | Other (Specify) |  |  | Other (Specify) |  |  | Other (Specify) |  | | Other (Specify) |  |  |
| Other (Specify) |  |  | Other (Specify) |  |  | Other (Specify) |  |  | Other (Specify) |  | | Other (Specify) |  |  |
| Other (Specify) |  |  | Other (Specify) |  |  | Other (Specify) |  |  | Other (Specify) |  | | Other (Specify) |  |  |
| Other (Specify) |  |  | Other (Specify) |  |  | Other (Specify) |  |  | Other (Specify) |  | | Other (Specify) |  |  |

In the next question (Q4) you are presented with the same list of names of people involved in CHPS implementation in Ghana in this district from the national, regional, district, Sub-district and CHPS levels; you are asked to rate your interactions with each person.

*[Note: Please add any other name (Name, title, facility) that is not listed]*

|  | **Q4: How often do you communicate with these national level persons on any issue related to CHPS?** | | | | | | |
| --- | --- | --- | --- | --- | --- | --- | --- |
|  | 0 = Never | 1 = Once a quarter | 2 = Monthly | 3 = Weekly | 4 = Daily | 99 = I don’t know the person | |
|  | Your answers *[Please mark with the corresponding number in each cell]* | | | | | | |
| **National level** |  | | | | | | **Q4** |
|  | Name 1 | | | | | |  |
|  | Name 2 | | | | | |  |
|  | Name 3 … | | | | | |  |
|  |  | | | | | |  |
|  | Other (Name, title, role) | | | | | |  |
|  | Other (Name, title, role) | | | | | |  |

|  | **Q4: How often do you communicate with these regional-level persons on any issues related to CHPS?** | | | | | | |
| --- | --- | --- | --- | --- | --- | --- | --- |
|  | 0 = Never | 1 = Once a quarter | 2 = Monthly | 3 = Weekly | 4 = Daily | 99 = I don’t know the person | |
|  | Your answers *[Please mark with the corresponding number in each cell]* | | | | | | |
| **Regional level** |  | | | | | | **Q4** |
|  | Name 1 | | | | | |  |
|  | Name 2 | | | | | |  |
|  | Name 3 … | | | | | |  |
|  |  | | | | | |  |
|  | Other (Name, title, role) | | | | | |  |
|  | Other (Name, title, role) | | | | | |  |

|  | **Q4: How often do you communicate with each of these district-level persons regarding CHPS issues?** | | | | | | |
| --- | --- | --- | --- | --- | --- | --- | --- |
|  | 0 = Never | 1 = Once a quarter | 2 = Monthly | 3 = Weekly | 4 = Daily | 99 = I don’t know the person | |
|  | Your answers *[Please mark with the corresponding number in each cell]* | | | | | | |
| **District level** |  | | | | | | **Q4** |
|  | Name 1 | | | | | |  |
|  | Name 2 | | | | | |  |
|  | Name 3 … | | | | | |  |
|  |  | | | | | |  |
|  | Other (Name, title, role) | | | | | |  |
|  | Other (Name, title, role) | | | | | |  |

| **Sub-district level** | **Q4: How often do you communicate with each of these sub-district level persons on CHPS issues?** | | | | | | |
| --- | --- | --- | --- | --- | --- | --- | --- |
|  | 0 = Never | 1 = Once a quarter | 2 = Monthly | 3 = Weekly | 4 = Daily | 99 = I don’t know the person | |
|  | Your answers *[Please mark with the corresponding number in each cell]* | | | | | | |
|  |  | | | | | | **Q4** |
|  | Name 1 | | | | | |  |
|  | Name 2 | | | | | |  |
|  | Name 3 … | | | | | |  |
|  |  | | | | | |  |
|  | Other (Name, title, role) | | | | | |  |
|  | Other (Name, title, role) | | | | | |  |

| **CHPS/Community level** | **Q4: How often do you communicate with each of these community-level persons regarding CHPS issues?** | | | | | | |
| --- | --- | --- | --- | --- | --- | --- | --- |
|  | 0 = Never | 1 = Once a quarter | 2 = Monthly | 3 = Weekly | 4 = Daily | 99 = I don’t know the person | |
|  | Your answers *[Please mark with the corresponding number in each cell]* | | | | | | |
|  |  | | | | | | **Q4** |
|  | Name 1 | | | | | |  |
|  | Name 2 | | | | | |  |
|  | Name 3 … | | | | | |  |
|  |  | | | | | |  |
|  | Other (Name, title, role) | | | | | |  |
|  | Other (Name, title, role) | | | | | |  |

**Part Two: Background**

1. Sex of Respondent

| - Female | 1 |
| --- | --- |
| - Male | 2 |

2. What is your age……………….

1. How long have you been involved in CHPS implementation in this district? …check the use district in this……………………………
2. What is your current position in the CHPS implementation? (*Mark with X*) *[Choose all that apply]*

| - National CHPS Coordinator |  |
| --- | --- |
| - Director General, GHS |  |
| - Deputy Director General, GHS |  |
| - Director Public health |  |
| - Director Health promotion division |  |
| - Director, Family health |  |
| - Director PPME, GHS |  |
| - Director Human Resource, GHS |  |
| - District health insurance officers |  |
| - CHPS coordinator |  |
| - District Director-HS |  |
| - Public Health Nurse |  |
| - Disease Control Officer |  |
| - Health promotion officer |  |
| - Health information officer |  |
| - Sub-district leader |  |
| - Field Technicians /Disease Control Officer |  |
| - CHPS in-charge |  |
| - Community health nurse /midwife / officer |  |
| - Community Health volunteer |  |
| - Development partner |  |
| - CEO (CSO, NGO etc) |  |
| - Other (Please specify) |  |
| - Other (Please specify) |  |
| - Other (Please specify) |  |
| - Other (Please specify) |  |

1. How long have you worked in this position (Q3)? ………………………………..
